# Chikungunya Virus Mutation, Indonesia, 2011

**DOI:** 10.3201/eid2102.141121

**Published:** 2015-02

**Authors:** Masri Sembiring Maha, Ni Ketut Susilarini, Nur Ika Hariastuti, Subangkit

**Affiliations:** National Institute of Health Research and Development, Jakarta, Indonesia

**Keywords:** **c**hikungunya, ECSA, A226V, mutation, Indonesia, viruses

**To the Editor:** Chikungunya virus (CHIKV) is a single-stranded, positive-sense RNA virus of ≈11.8 kb molecules (*1*) belonging to the family *Togaviridae* and genus *Alphavirus*. Genotypes of CHIKV include Asian, East/Central/South African (ECSA), and West African. CHIKV is endemic to Africa, southern Asia, and Southeast Asia and frequently causes debilitating but nonfatal illness.

CHIKV attracted global attention when a large epidemic on Réunion Island in 2005–2006 spread rapidly to other parts of the world (*1*). The predominant strain during this epidemic was the ECSA genotype with the A226V mutation of the E1 protein (*2*), the transmission of which is reported to be facilitated by *Aedes albopictus* mosquitoes (*3*). The ECSA genotype has been reported to circulate in Southeast Asia, including Malaysia, but not in Indonesia (*4*). Concern about circulating ESCA strains triggered alerts in 2009, when the Indonesian Ministry of Health reported an increasing number of chikungunya cases (3,529 cases in 2008, 83,756 in 2009) (*5*). However, only Asian genotypes were detected (*4*). We investigated recent outbreaks of CHIKV in Indonesia and genotypes of associated CHIKV strains.

After chikungunya outbreaks were reported from 6 districts in Indonesia (Tangerang, Karang Anyar, Ngawi, Jembrana, Mataram, and Kubu Raya), a team from the National Institute of Health and Research Development, Indonesian Ministry of Health, conducted field investigations from April through October 2011. This study received institutional review board approval (KE.01.06/EC/373/2011).

Serum specimens from persons with fever >38°C who provided signed informed consent were tested at the Virology Laboratory, Center for Biomedical and Basic Technology of Health, National Institute of Health Research and Development, in Jakarta. Molecular examination by reverse transcription PCR (RT-PCR) of acute-phase serum specimens, selective for the E1 gene, was performed as previously described (*6*). Amplicons (330 bp) were sequenced for confirmation. The entire E1 gene of 2 identified ECSA genotypes was sequenced (*7*). A cladogram was created by using MEGA version.6.06 and the neighbor-joining method (*8*). The strength of the cladogram was estimated by bootstrap analyses that used 1,000 random samplings. To determine the circulating genotype of CHIKV in Indonesia, we compared these results with other reference sequences in GenBank. 

RT-PCR confirmed CHIKV in 28 (26%) of 109 samples from 5 districts: 12 (50%) in Mataram, 8 (47%) in Jembrana, 2 (40%) in Tangerang, 4 (21%) in Ngawi, and 2 (9%) in Kubu Raya. No samples from Karang Anyer were positive for CHIKV. Sequencing analysis revealed the A226V mutant (alanine to valine) ECSA genotype in 2 (7%) specimens (GenBank accession nos. KJ729851, KH729852) and the Asian genotypes (KJ729829–50, KJ729853–56) in 26 (93%) specimens. The Asian genotypes were closely related to those of CHIKV isolated from East Kalimantan, Bandung, Malaysia, and India (Figure).

**Figure:**
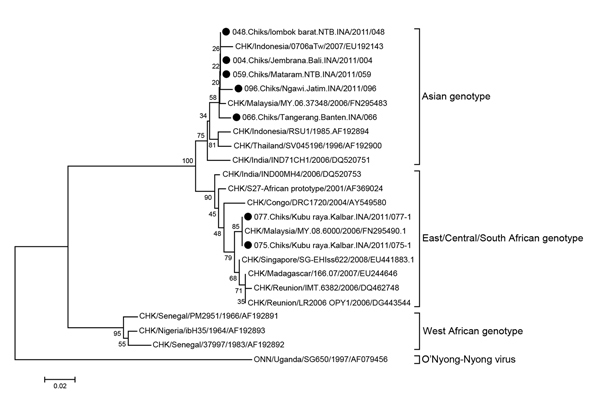
Cladogram of chikungunya virus, Asian and East/Central/South African genotypes, from chikungunya fever outbreaks in 6 districts in Indonesia, April–October 2011. Black dots indicate samples from patients. Scale bar indicates nucleotide substitutions per site. CHK, chikungunya; Chiks, chikungunya.

The 2 cases associated with the A226V mutant ECSA genotype occurred in October 2011 in the Kubu Raya district, West Kalimantan, near the Malaysia border. Because both patients had no history of travel to Malaysia, where outbreaks involving the ECSA genotype had been reported, this finding demonstrates the emergence of the CHIKV A226V ECSA genotype in Indonesia. The 2008 nationwide outbreak of chikungunya in Malaysia proved that A226V mutation enhances transmissibility of CHIKV by *Ae. albopictus* mosquitoes (*9*). Population movement from this region might contribute to the spread of this virus to Indonesia, which is a concern because of the higher transmissibility of the mutated ECSA strain through the *Ae. albopictus* mosquito vector, which is prevalent throughout Indonesia.

That ECSA genotypes were not found in other districts during this investigation would suggest that this strain was not the source of the 2008–2009 outbreaks in Indonesia, although this suggestion is by no means certain. The predominance of the Asian genotypes suggests endemicity of similar CHIKV strains.

A limitation of this study was the lack of serologic assays to confirm CHIKV infections, especially for those who sought care late after onset of illness. Because of the lack of reliable serologic assays with high sensitivity, some cases deemed by RT-PCR to be CHIKV negative might have been clinical cases of CHIKV infection (*10*). 

Thus, the A226V ECSA genotype of CHIKV was circulating in Indonesia 5 years after the global pandemic and 2–3 years after the emergence of this strain from other Southeast Asian countries (*4*). Sensitive serology-based assays and rapid tests for different operational settings are needed, especially in those areas without molecular diagnostic capabilities. Also needed is surveillance of CHIKV throughout Indonesia so that health policy makers can have comprehensive data on the molecular epidemiology and prevalence of CHIKV infection. In addition, studies of CHIKV transmission by different vectors as well as virus and vector interactions are needed to provide an understanding of the emergence of the mutant strain across the region and to assist strategies for vector control and disease prevention and control.

## References

[1] Powers AM. Chikungunya. Clin Lab Med. 2010;30:209–19. 10.1016/j.cll.2009.10.003 20513548

[2] Schuffenecker I, Iteman I, Michault A, Murri S, Frangeul L, Vaney MC, Genome microevolution of chikungunya viruses causing the Indian Ocean outbreak. PLoS Med. 2006;3:e263. 10.1371/journal.pmed.0030263 16700631 PMC1463904

[3] Schwartz O, Albert ML. Biology and pathogenesis of chikungunya virus. Nat Rev Microbiol. 2010;8:491–500. 10.1038/nrmicro2368 20551973

[4] Kosasih H, de Mast Q, Widjaja S, Sudjana P, Antonjaya U, Ma’roef C, Evidence for endemic chikungunya virus infections in Bandung, Indonesia. PLoS Negl Trop Dis. 2013;7:e2483. 10.1371/journal.pntd.0002483 24205417 PMC3812099

[5] Departemen Kesehatan Republik Indonesia. Pusdatin. Profil Kesehatan Indonesia 2009. Jakarta (Indonesia): The Ministry; 2010:47.

[6] M Naresh Kumar CV. Anthony Johnson AM, R Sai Gopal DV. Molecular characterization of chikungunya virus from Andhra Pradesh, India & phylogenetic relationship with Central African isolates. Indian J Med Res. 2007;126:534–40 . 18219080

[7] Ng LC, Tan LK, Tan CH, Tan SS, Hapuarachchi HC, Pok KY, Entomologic and virologic investigation of chikungunya, Singapore. Emerg Infect Dis. 2009;15:1243–9. 10.3201/eid1508.081486 19751586 PMC2815960

[8] Tamura K, Stecher G, Peterson D, Filipski A, Kumar S. MEGA6: Molecular Evolutionary Genetics Analysis version 6.0. Mol Biol Evol. 2013;30:2725–9. 10.1093/molbev/mst197 24132122 PMC3840312

[9] Sam IC, Chan YF, Chan SY, Loong SK, Chin HK, Hooi PS, Chikungunya virus of Asian and Central/East African genotypes in Malaysia. J Clin Virol. 2009;46:180–3. 10.1016/j.jcv.2009.07.016 19683467

[10] Yap G, Pok KY, Lai YL, Hapuarachchi HC, Chow A, Leo YS, Evaluation of chikungunya diagnostic assays: differences in sensitivity of serology assays in two independent outbreaks. PLoS Negl Trop Dis. 2010;4:e753. 10.1371/journal.pntd.0000753 20651930 PMC2907414

